# Inferior Frontal Gyrus Activation Underlies the Perception of Emotions, While Precuneus Activation Underlies the Feeling of Emotions during Music Listening

**DOI:** 10.1155/2015/529043

**Published:** 2015-10-04

**Authors:** Ken-ichi Tabei

**Affiliations:** ^1^Department of Dementia Prevention and Therapeutics, Graduate School of Medicine, Mie University, Tsu 514-8507, Japan; ^2^Department of Neurology, Graduate School of Medicine, Mie University, Tsu 514-8507, Japan

## Abstract

While music triggers many physiological and psychological reactions, the underlying neural basis of perceived and experienced emotions during music listening remains poorly understood. Therefore, using functional magnetic resonance imaging (fMRI), I conducted a comparative study of the different brain areas involved in perceiving and feeling emotions during music listening. I measured fMRI signals while participants assessed the emotional expression of music (perceived emotion) and their emotional responses to music (felt emotion). I found that cortical areas including the prefrontal, auditory, cingulate, and posterior parietal cortices were consistently activated by the perceived and felt emotional tasks. Moreover, activity in the inferior frontal gyrus increased more during the perceived emotion task than during a passive listening task. In addition, the precuneus showed greater activity during the felt emotion task than during a passive listening task. The findings reveal that the bilateral inferior frontal gyri and the precuneus are important areas for the perception of the emotional content of music as well as for the emotional response evoked in the listener. Furthermore, I propose that the precuneus, a brain region associated with self-representation, might be involved in assessing emotional responses.

## 1. Introduction

Music has strong emotional effects on listeners. When investigating the effect of music on emotions, it is necessary to consider the felt emotion separately from the perceived emotion [1, 2]. When we listen to music, various emotions are induced in our mind. On the other hand, we can objectively appreciate the emotions expressed in the music independently of our own emotions. The former is referred to as felt emotion, which is an emotional response evoked by music in listeners. The latter is referred to as perceived emotion, which is an emotional quality detected by the listeners of music [1, 2]. Hunter and Schellenberg [3] argued about perceived emotion and felt emotion; when a listener is in a happy mood, it is possible for them to maintain that happy feeling even when they are listening to sad music, which they perceive as being sad. However, if the music is associated with an unpleasant past experience, a disagreeable feeling could be evoked in the listener even when they recognize the music as expressing a happy feeling. Together with previous findings, it seems that feeling and perception ratings are correlated, although perceived emotion tends to be stronger than felt emotion [4–7]. Therefore, they advocated that it was necessary to separate these two types of loci.

As for the relationship between these two types of perception, past studies using psychological measurements have reported a correlation between the perceived emotion and felt emotion [4–7]. However, compared to the perceived emotion, the felt emotion includes various factors, such as recalling memories associated with the music [7]. Therefore, when the mean values were calculated from the listeners' assessments of the emotional expression of music and their assessments of their own emotional response, the absolute value of the rating scales of the listeners' emotional response tended to be lower than that of the emotional expression of the music. This finding suggests that while listeners can easily perceive the emotional expression intended by the composer, listeners may not necessarily experience the emotional response that the composer intended to convey [3, 7].

Past neuropsychological studies of brain-damaged patients have suggested that the perceived emotion and felt emotion while listening to music may differ. Satoh et al. [8] reported a patient with damage to the parietal lobe in his right cerebral hemisphere who had selective impairment of felt emotion while listening to music; however, the patient's intellectual function, memory, constructional ability, and perception of the emotional expression of the music remained normal. Similar impairments have also been reported for a patient with damage to the temporal and parietal lobes in the right hemisphere [9] and a patient with a lesion centering on the insular cortex in the left hemisphere, which widely included the frontal lobe and amygdala [10]. On the other hand, there are also patients who show the opposite symptoms [11, 12]. Matthews et al. [11] reported a case of neurodegenerative disease with auditory agnosia in which the auditory agnosia was accompanied by impaired perception of the emotional expression of music and musical components such as pitch, while the emotional response when listening to music was maintained.

Thus, past psychological and brain injury studies have demonstrated that the perceived emotion and felt emotion while listening to music could be separable loci, which suggests that they may involve different localizations in the brain.

Recently, there have been brain function imaging studies that have attempted to visualize human brain activity while listening to music. Several studies pertaining to the perceived emotion [13–15] and felt emotion [16–20] while listening to music have been conducted. These studies have reported that when participants are assessing the emotional expression of music, activity occurs in the medial frontal gyrus, superior frontal gyrus, middle frontal gyrus, and cingulate gyrus; and when participants are assessing their emotion felt to music, activity occurs in the ventral striatum, midbrain, amygdala, orbitofrontal cortex, and ventral prefrontal area.

The purpose of these studies was to determine the areas of the brain that are involved in cognitive processing by assessing perceived emotion alone or felt emotion alone while listening to music. However, as seen in the results of psychological and brain injury studies, it is obvious that there is some interaction between the cognitive processes of emotion felt to and emotional expression of music, which suggests that there are both common and independent areas in the brain related to the processing of these two emotional loci. Therefore, in order to clarify the whole process, it is essential to compare directly both the emotion felt and emotional expression and to investigate the brain localizations related to these two emotional loci. The objectives of this study were as follows: (1) to use functional magnetic resonance imaging (fMRI) to measure brain activity while a participant is assessing their emotion felt and the emotional expression evoked by the music and (2) to compare the areas of brain activity involved in these respective assessments. Many common brain mechanisms exist for the assessment of emotional response and of emotional expression while listening to music. However, the locus differs between the recognition of one's own emotion (felt emotion) and the understanding of what the music is expressing (perceived emotion). Therefore, it is predicted that the information processing of the two respective loci will involve independent brain localizations.

## 2. Materials and Methods

### 2.1. Participants

Seventeen healthy right-handed undergraduate students majoring in music (10 women, 7 men; mean age 21.4 years, SD = 2.0) participated in this study. Participants provided written informed consent before the experiment in accordance with the Declaration of Helsinki. The study was approved by the Ethics Committee of Nihon University.

### 2.2. Stimulus Selection

Four hundred and fifty-two healthy volunteers (311 men and 141 women), between 18 and 29 years of age (mean age 19.3 years, SD = 1.4), performed one of two rating tasks. None of the volunteers participated in the subsequent fMRI study. A set of 56 musical stimuli (16 happy, 16 sad, 16 scary, and 16 peaceful stimuli from Vieillard et al. [21]) were copied onto two CDs in a random order, with 6 s of silence between excerpts. Two groups of listeners were defined based on the type of ratings they were requested to provide. Each excerpt was presented only once. In the felt emotion task, 232 listeners judged to what extent they experienced each of the four emotions labeled as happy, sad, scary, and peaceful. In the perceived emotion task, 220 listeners judged to what extent they perceived each of the four emotions labeled as happy, sad, scary, and peaceful in each excerpt. For each labeled emotion, listeners gave a rating on a 10-point scale from 0 (*absent*) to 9 (*present*). The listeners had been previously informed that they had to provide a rating for the four emotion labels. I calculated the average scale of each stimulus. I used only happy and sad stimuli for the fMRI experiment because it was easy for participants to differentiate happy and sad stimuli. As a result, 12 happy (g01, g03, g04, g07, g10, and g13) and sad (t04, t06, t08, t10, t13, and t14) [21, APPENDIX 2] musical stimuli were selected for the fMRI experiment from the six top average stimuli from the perceived and felt emotion tasks. The happy stimuli were written in a major mode at an average tempo of 137 metronome markings (MM; range: 92–196) [21]. The sad stimuli were written in a minor mode at an average slow tempo of 46 MM (range: 40–60) [21]. The average stimuli duration was 12.4 s (range: 9.2–16.4 s) [21].

### 2.3. Tasks

The stimuli were presented through stereo headphones and the instructions were presented using a projector in the fMRI scanner. The participants performed three tasks. In the felt emotion task, they judged whether they felt happy, sad, or neutral emotions and pressed the button assigned to each emotional response. I asked participants “Please indicate which of the three emotions you experience in each excerpt.” In the perceived emotion task, they judged whether the stimulus expressed happy, sad, or neutral feelings and pressed the button assigned to each emotional quality. I asked participants “Please indicate which of the three emotions you recognize in each excerpt.” The participants performed the three-forced-choice judgment in each task. In the passive listening task, the participants listened to the stimulus and then pressed a button. A block design was used. Each task condition lasted for 60 s with instruction slides followed by a baseline period (32 s). In each task block, three musical stimuli were presented in a random order every 20 s. Participants performed each task six times in a recording session.

### 2.4. fMRI Measurements

The MRI scans were obtained using a 1.5-Tesla MR scanner (Siemens Symphony). Functional images were obtained using a T2^*∗*^-weighted gradient-echo planar imaging sequence (40 horizontal slices, repetition time (TR) = 4,000 ms, echo time (TE) = 50 ms, slice thickness = 3 mm, gapless, and field of view (FOV) = 192 mm, 64 × 64 matrix). Additionally, a T1-weighted anatomical image was obtained for each participant (TR = 2,200 ms, TE = 3.93 ms, FA = 15°, TI = 1,100 ms, 1 mm^3^ voxel, and FOV = 256 mm).

### 2.5. fMRI Data Analysis

Data analysis was performed using SPM5 (Wellcome Department of Imaging Neuroscience, London, UK). The functional images were realigned to the first image to correct for movement-related effects, coregistered to the anatomical image, normalized to the Montreal Neurological Institute brain template, and spatially smoothed with an isotropic Gaussian kernel (full width at half maximum = 8 mm). I conducted voxelwise statistical analyses based on the general linear model. For the statistical model, a block design was modeled using the canonical hemodynamic response function and the temporal derivative, and low-frequency drifts were removed using a high-pass filter (128 s). For each participant, I computed contrasts for “perceived emotion task > baseline,” “felt emotion task > baseline,” and “passive listening task > baseline.” In addition, I also computed contrasts for “perceived emotion task > passive listening task,” “perceived emotion task > felt emotion task,” “felt emotion task > passive listening task,” and “felt emotion task > perceived emotion task.” In order to exclude the possibility of negative activations, I applied an inclusive mask using the contrast image of “perceived emotion task > baseline” (in the former two contrasts) and “felt emotion task > baseline” (in the latter two contrasts). A random-effects model was used for the group analysis (a voxel level after correction for false discovery rate *p* < 0.05 or cluster threshold *p* < 0.05 (corrected) and a voxel threshold *p* < 0.001 (uncorrected)).

## 3. Results

### 3.1. Perceived Emotion Is Stronger Than Felt Emotion across Participants

**Figure 1:**
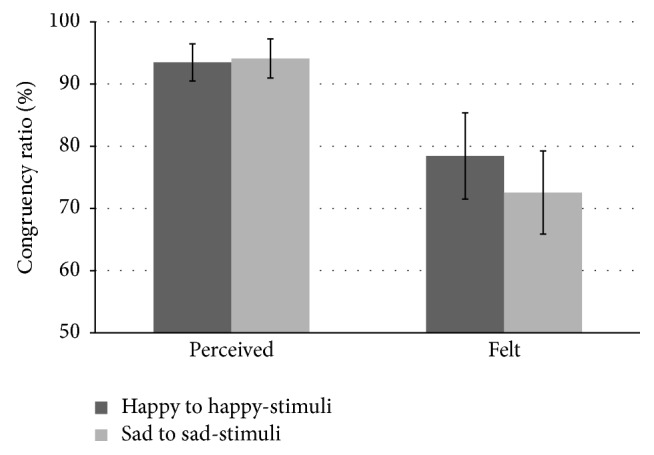
Congruency ratio of selected adjective to music categories. The bars represent standard deviation.

Each participant's judgment in the perceived emotion and felt emotion tasks was calculated as the congruency ratio of selected adjective to music category (i.e., happy to happy-stimuli and sad to sad-stimuli; Figure 1). In the perceived emotion task, the mean of the congruency ratio was 93.5% and 94.1% for happy to happy-stimuli and sad to sad-stimuli, respectively. In the felt emotion task, the mean of the congruency ratio was 78.4% and 72.5% for happy to happy-stimuli and sad to sad-stimuli, respectively. The effect of tasks on perceived versus felt emotion was tested by performing two-way analysis of variance with the congruency ratio of selected adjective to musical category for the task condition (perceived versus felt) and musical category (happy versus sad) as within-participants factors. A significant main effect of task (*F*(1,16) = 7.64, *p* < 0.05) was observed, with a higher congruency ratio in the perceived emotion condition than in the felt emotion condition. No significant main effect of musical category or interaction between task condition and musical category was found.

### 3.2. Perceived Emotions Activate the Bilateral Inferior Frontal Gyri, While Felt Emotions Activate the Precuneus

**Figure 2:**
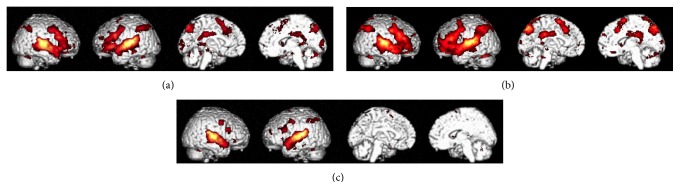
Cortical areas where the blood-oxygen-level dependent signals increased with the perceived emotion (a), felt emotion (b), and passive listening (c) tasks; voxel level after correction for false discovery rate (*p* < 0.05).

Using baseline subtraction analysis, I found that the perceived emotion task was associated with increased blood-oxygen-level dependent (BOLD) signals in areas of the frontal cortex (the inferior, middle, and superior frontal gyri and medial wall), auditory areas, the posterior parietal cortex, precuneus, parahippocampal gyrus, cingulate gyrus, right lentiform nucleus, left thalamus, and cerebellum (Figure 2(a)). The felt emotion task was associated with increased BOLD signals in areas of the frontal cortex (the inferior, middle, and superior frontal gyri and medial wall), auditory areas, the posterior parietal cortex, precuneus, cingulate gyrus, right lentiform nucleus, left thalamus, and cerebellum (Figure 2(b)). Meanwhile, the passive listening task was associated with increased BOLD signals in areas of the frontal cortex (inferior and middle frontal gyri), auditory areas, the posterior parietal cortex, and the cerebellum (Figure 2(c)).

**Figure 3:**
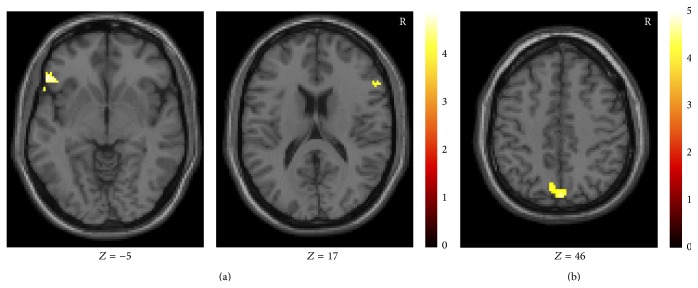
Cortical areas where the blood-oxygen-level dependent signals increased with perceived emotion > passive listening (a) and felt emotion > passive listening (b); cluster threshold *p* < 0.05 (corrected).

When I performed subtraction analysis with the passive listening task, the perceived emotion task was associated with increased BOLD signals in the bilateral inferior frontal gyri (Table 1(a); Figure 3(a)) and the felt emotion task was associated with increased BOLD signals in the precuneus (Table 1(b); Figure 3(b)). On the other hand, a direct comparison of the passive listening and the perceived emotion tasks and of the passive listening and the felt emotion tasks did not show significant activation. A direct comparison between the perceived and felt emotion tasks also did not show significant activation.

## 4. Discussion

In this study, brain activity was measured using fMRI while the participants were assessing the emotional expression of music and while they were assessing the emotional response they felt while listening. I compared the active brain areas that were involved in each of the assessments. I found that the study participants assessed sad stimuli as more “sad” and happy stimuli as more “happy” in the perceived emotion task than in the felt emotion task. Similar to previous studies [4–7], I confirmed that perceived emotion was assessed more reliably by the participants than emotional responses.

A comparison of brain activity in each of the tasks showed that the precuneus region was activated more strongly in the felt emotion task than in the passive listening task. In addition, greater activity in the bilateral inferior frontal gyri was observed during the perceived emotion task than during the passive listening task. The precuneus region is known to be the area responsible for “self-representation” and is activated during tasks involving self-referential judgments and tasks consisting of judgments from a first-person perspective [22]. In addition, previous work where only the listeners' emotional responses were assessed found that activity in the precuneus region was correlated with the emotional response [16]. Therefore, this study suggests that the precuneus region is concerned with evaluating one's own cognitive emotional changes during music listening.

Activity in the inferior frontal gyri increased during the perceived emotion task. Although there have been no previous reports on inferior frontal gyri activation during emotional assessment, previous studies have reported activity in the dorsolateral prefrontal cortex (Brodmann area 9) during the assessment of emotion expressions [23]. One reason for the paucity of reports may stem from the fact that previous studies have focused on the brain lateralization of the perception of emotional expressions of happy and sad music. However, the dorsolateral prefrontal cortex has been reported to be active during the judgment of tonality [24] and the detection of deviations from harmonic progressions [25], suggesting that this area might be involved in the processing of tonality in musical structures. The musical stimuli used in the current study also consisted of music involving major and minor tonalities and may account for the inferior frontal gyri activation.

## 5. Conclusion

In summary, the findings suggest that the bilateral inferior frontal gyri might be involved in assessing the emotional expression of music, while the precuneus processes the emotional responses evoked in the listener. Moreover, the fact that the precuneus region is likely responsible for self-representation implicates this brain area in the assessment of the emotional response to music.

## Figures and Tables

**(a):** 

Contrast	L/R	Area	Brodmann area (BA)	*Z* value	Talairach coordinates (mm)		
					*X*	*Y*	*Z*
Perceived > passive	Right	Inferior frontal gyrus	BA 45	3.63	53	28	8
				3.45	55	26	17
	Left	Inferior frontal gyrus	BA 47	3.78	−53	27	−5
				3.49	−55	17	−3
				3.39	−46	31	0

**(b):** 

Contrast	L/R	Area	Brodmann area (BA)	*Z* value	Talairach coordinates (mm)		
					*X*	*Y*	*Z*
Felt > passive	Right	Precuneus	BA 7	3.69	2	−75	46
	Left	Precuneus	BA 7	3.87	−8	−66	35
				3.32	−4	−70	44

## References

[1] Gabrielsson A. (2002). Emotion perceived and emotion felt: same or different?. *Musicae Scientiae*.

[2] Schubert E. (2013). Emotion felt by the listener and expressed by the music: literature review and theoretical perspectives. *Frontiers in Psychology*.

[3] Hunter P. G., Schellenberg E. G., Riess Jones M., Fay R. R., Popper A. (2010). Music and emotion. *Music Perception*.

[4] Evans P., Schubert E. (2008). Relationships between expressed and felt emotions in music. *Musicae Scientiae*.

[5] Kallinen K., Ravaja N. (2006). Emotion perceived and emotion felt: same and different. *Musicae Scientiae*.

[6] Schubert E. (2007). Locus of emotion: the effect of task order and age on emotion perceived and emotion felt in response to music. *Journal of Music Therapy*.

[7] Schubert E. (2007). The influence of emotion, locus of emotion and familiarity upon preference in music. *Psychology of Music*.

[8] Satoh M., Nakase T., Nagata K., Tomimoto H. (2011). Musical anhedonia: selective loss of emotional experience in listening to music. *Neurocase*.

[9] Mazzoni M., Moretti P., Pardossi L., Vista M., Muratorio A., Puglioli M. (1993). A case of music imperception. *Journal of Neurology Neurosurgery and Psychiatry*.

[10] Griffiths T. D., Warren J. D., Dean J. L., Howard D. (2004). ‘When the feeling's gone’: a selective loss of musical emotion. *Journal of Neurology, Neurosurgery and Psychiatry*.

[11] Matthews B. R., Chang C.-C., de May M., Engstrom J., Miller B. L. (2009). Pleasurable emotional response to music: a case of neurodegenerative generalized auditory agnosia. *Neurocase*.

[12] Peretz I., Gagnon L. (1999). Dissociation between recognition and emotional judgements for melodies. *Neurocase*.

[13] Blood A. J., Zatorre R. J., Bermudez P., Evans A. C. (1999). Emotional responses to pleasant and unpleasant music correlate with activity in paralimbic brain regions. *Nature Neuroscience*.

[14] Brown S., Martinez M. J., Parsons L. M. (2004). Passive music listening spontaneously engages limbic and paralimbic systems. *NeuroReport*.

[15] Menon V., Levitin D. J. (2005). The rewards of music listening: response and physiological connectivity of the mesolimbic system. *NeuroImage*.

[16] Blood A. J., Zatorre R. J. (2001). Intensely pleasurable responses to music correlate with activity in brain regions implicated in reward and emotion. *Proceedings of the National Academy of Sciences of the United States of America*.

[17] Koelsch S., Fritz T., Cramon D. Y. V., Müller K., Friederici A. D. (2006). Investigating emotion with music: an fMRI study. *Human Brain Mapping*.

[18] Baumgartner T., Esslen M., Jäncke L. (2006). From emotion perception to emotion experience: emotions evoked by pictures and classical music. *International Journal of Psychophysiology*.

[19] Baumgartner T., Lutz K., Schmidt C. F., Jäncke L. (2006). The emotional power of music: how music enhances the feeling of affective pictures. *Brain Research*.

[20] Mitterschiffthaler M. T., Fu C. H. Y., Dalton J. A., Andrew C. M., Williams S. C. R. (2007). A functional MRI study of happy and sad affective states induced by classical music. *Human Brain Mapping*.

[21] Vieillard S., Peretz I., Gosselin N., Khalfa S., Gagnon L., Bouchard B. (2008). Happy, sad, scary and peaceful musical excerpts for research on emotions. *Cognition and Emotion*.

[22] Cavanna A. E., Trimble M. R. (2006). The precuneus: a review of its functional anatomy and behavioural correlates. *Brain*.

[23] Khalfa S., Schon D., Anton J.-L., Liégeois-Chauvel C. (2005). Brain regions involved in the recognition of happiness and sadness in music. *NeuroReport*.

[24] Mizuno T., Sugishita M. (2007). Neural correlates underlying perception of tonality-related emotional contents. *NeuroReport*.

[25] Steinbeis N., Koelsch S. (2008). Shared neural resources between music and language indicate semantic processing of musical tension-resolution patterns. *Cerebral Cortex*.

